# Synthesis and Pro-Apoptotic Activity of Novel Glycyrrhetinic Acid Derivatives

**DOI:** 10.1002/cbic.201000618

**Published:** 2011-02-15

**Authors:** Evgeniya B Logashenko, Oksana V Salomatina, A V Markov, Dina V Korchagina, Nariman F Salakhutdinov, Genrikh A Tolstikov, Valentin V Vlassov, Marina A Zenkova

**Affiliations:** [a] Institute of Chemical Biology and Fundamental Medicine, Siberian Branch, Russian Academy of Sciences8, Lavrent'ev avenue, 630090 Novosibirsk (Russian Federation) Fax: (+7) 383-36-35-160 E-mail: marzen@niboch.nsc.ruevg_log@niboch.nsc.ru; [b] Vorozhtsov Institute of Organic Chemistry, Siberian Branch, Russian Academy of Sciences9, Lavrent'ev avenue, 630090 Novosibirsk (Russian Federation)

**Keywords:** antitumor agents, apoptosis, biological activity, glycyrrhetinic acid derivatives, medicinal chemistry

## Abstract

Triterpenoids are used for medicinal purposes in many countries. Some, such as oleanolic and glycyrrhetinic acids, are known to be anti-inflammatory and anticarcinogenic. However, the biological activities of these naturally occurring molecules against their particular targets are weak, so the synthesis of new synthetic analogues with enhanced potency is needed. By combining modifications to both the A and C rings of 18βH-glycyrrhetinic acid, the novel synthetic derivative methyl 2-cyano-3,12-dioxo-18βH-olean-9(11),1(2)-dien-30-oate was obtained. This derivative displays high antiproliferative activity in cancer cells, including a cell line with a multidrug-resistance phenotype. It causes cell death by inducing the intrinsic caspase-dependent apoptotic pathway.

## Introduction

Organic molecules synthesized by plants constitute a rich reservoir of biologically active compounds. For centuries extracts from various plants have been extensively used in traditional medicines for the treatment of a wide variety of human ailments; even today, many cultures still employ them directly for medicinal proposes.[1–4] Among the classes of recognized therapeutically useful products, pentacyclic triterpeniods have been studied intensively for their diverse biological, pharmacological, and medicinal activities, which are similar to those of retinoids and steroids.[5, 6] However, these triterpeniods exhibit only weak effects on the biological activity of their molecular targets; therefore these compounds have been used as building blocks for the synthesis of more active analogues.[6]

**Scheme 1:**
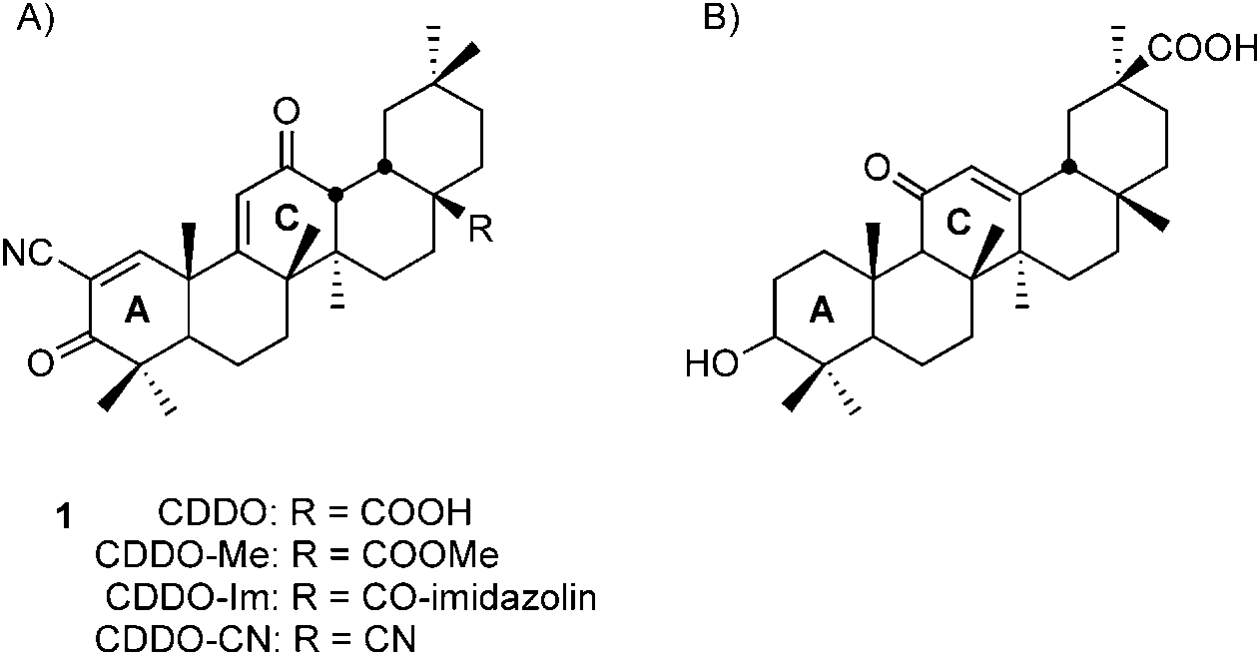
A) Structure of CDDO and its structurally related analogues. B) Structure of glycyrrhetinic acid.

Oleanolic acid, an abundantly occurring triterpene, has been converted into 2-cyano-3,12-dioxooleana-1,9-dien-28-oic acid (CDDO) and other structurally related analogues (CDDO-Me, CDDO-Im, CDDO-CN; Scheme 1A).[7–9] All of these synthetic derivatives were reported to display various bioactivities: cytoprotection, cancer cell growth inhibition, apoptosis induction, and inhibition of the production of NO induced by INF-γ in mouse macrophages.[7–12] CDDO and CDDO-Me are currently in clinical trials for cancer treatment, and have been shown to effectively suppress the growth of a broad spectrum of solid and hematologic cancer cell types, both in vitro and in mouse models bearing xenografted human tumors.[9–12] During the development of CDDO, it was found that the 2-cyano-1-en-3-one in ring A, and the 9(11)-en-12-one in ring C are essential for the biological activity of CDDO and its analogues.[13–15]

18βH-Glycyrrhetinic acid (Scheme 1B), the aglycon of glycyrrhizin, is abundant in licorice root (*Glycyrrhiza glabra* and *Glycyrrhiza uralensis Fischer*). The glycyrrhizin content in triterpene extracts from licorice root amounts to 90 %. Recent reviews have described the wide spectrum of glycyrrhetinic acid bioactivity, such as anti-inflammatory, antiviral, hepatoprotective, antitumor, and immunomodulatory activities.[16, 17] Several studies have reported that glycyrrhizin and glycyrrhetinic acid have moderate cytotoxic and apoptotic effects on cancer cells, although most reported only moderate or low potency.

In attempts to prepare more-potent analogues of glycyrrhetinic acid, we synthesized compounds **2**–**12,** similar to CDDO-Me (**1**), by introducing modification at both rings A and C (Scheme 1A). We investigated the effects of the novel derivatives on the growth of human cancer cells, and we identified methyl 2-cyano-3,12-dioxo-18βH-olean-9(11),1(2)-dien-30-oate **12** as a compound displaying significant antiproliferative activity toward cancer cells; the other glycyrrhetinic-acid derivatives did not display this activity. We compared **12** and CDDO-Me on several cell lines under the same conditions, and we showed that IC_50_ was lower for **12** than CDDO-Me for all cell lines. Compound **12** induced cell-cycle arrest, the translocation of phosphatidylserine to the cell surface, and fragmentation of the nucleus. It also caused a dramatic dissipation of the mitochondrial potential, and induced activation of the caspase cascade; these effects were more pronounced for **12** than for CDDO-Me. The data indicate that **12** induces the death of cancer cells by the intrinsic caspase-dependent apoptosis pathway.

## Results and Discussion

### Chemical synthesis

**Scheme 2:**
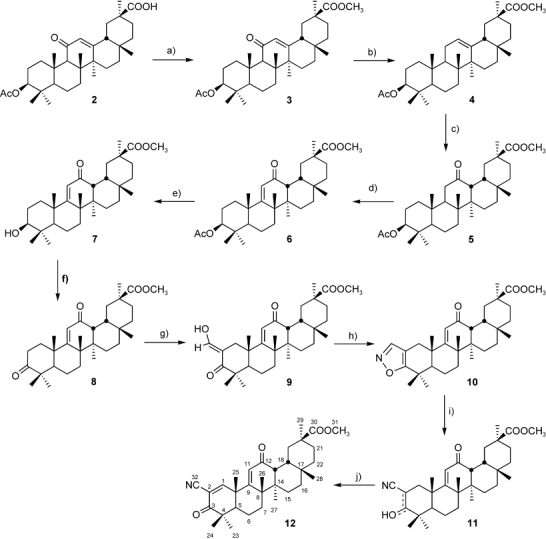
Synthesis of methyl 2-cyano-3,12-dioxo-18β*H*-olean-9(11),1(2)-dien-30-oate (**12**). a) CH_2_N_2_, MeOH-Et_2_O (0°C to RT), 89 %; b) Zn (powder), HCl (conc.), dioxane (5–10°C), 77 %; c) H_2_O_2_, AcOH (80°C), 96 %; d) Br_2_, AcOH (80°C), 75 %; e) KOH, MeOH (reflux), 90 %; f) Jones reagent, acetone (RT), 94 %; g) HCOOEt, NaOMe, benzene (RT), 95 %; h) NH_2_OH⋅HCl, EtOH-H_2_O (reflux), 73 %; i) NaOMe, MeOH-Et_2_O (0°C to RT), 100 %; j) 2,3-dichloro-5,6-dicyano-1,4-benzoquinone (DDQ), benzene (reflux), 61 %.

The reaction sequence to introduce the 2-cyano-1-en-3-one and 9(11)-en-12-one in A and C rings of glycyrrhetinic acid is shown in Scheme 2.

18βH-Glycyrrhetinic acid acetate **2,** obtained from a licorice extract, was used as the starting material. Compound **2** was esterified at 0°C with ethereal diazomethane to give methyl glycyrrhetinate acetate **3**, which was reduced by Zn/HCl in dioxane at 5–10°C. The resulting methyl ester of 11-deoxoglycyrrhetinic acid acetate **4** was converted into 12-oxo derivative **5** by treating with hydrogen peroxide in acetic acid at 80°C. The formation of the 9,11-double bond was achieved by bromination–dehydrobromination of ketone **5** with bromine in acetic acid at 80°C. Finally the 9(11)-en-12-one moiety in the C ring was obtained. Deprotection of acetate group by KOH in methanol (reflux) freed the 3-hydroxy group, then Jones oxidation gave the ketone **8**. Subsequent formylation at C^2^ was performed by condensation with HCO_2_Et/NaOMe in benzene, and the resulting hydroxymethylene derivative **9** was cyclized into isoxazole **10** by reacting with hydroxylamine hydrochloride in aqueous ethanol (reflux). Opening of the isoxazole ring at the N–O bond was promoted by NaOMe routinely to deliver the 2-cyano group in **11**. The new 1,2 double bond was formed by dehydrogenation with 2,3-dichloro-5,6-dicyano-1,4-benzoquinone (DDQ) in benzene (reflux) to complete the synthesis of the 2-cyano-1-en-3-one moiety in A ring in **12**.

It should be noted that the synthesis scheme for our end-product **12** has been described by Chadalapaka et al.[18] Our investigations were conducted independently and in parallel. In addition to the synthesis scheme, a detailed description of the synthesis and physicochemical properties of the end product (and of the intermediates) is presented in this work (see the Experimental Section).

### Biological studies

*Cell-viability inhibition:* Inhibitors of cell growth are potentially useful as chemopreventive and chemotherapeutic agents. The in vitro cytotoxicity of the novel derivatives of glycyrrhetinic acid against human epidermoid cancer cell-line KB-3-1 was determined by using the MTT assay, a colorimetric technique for the determination of cell viability which was developed for the initial stages of drug screening, The assay quantifies the reduction of the yellow tetrazole 3-(4,5-Dimethylthiazol-2-yl)-2,5-diphenyltetrazolium bromide (MTT) to its purple formazan derivative by mitochondrial dehydrogenase. It assumes cell viability to be proportional to the production of formazan, and thus low IC_50_ values imply high cytotoxicity or antiproliferation activity.

**Figure 1:**
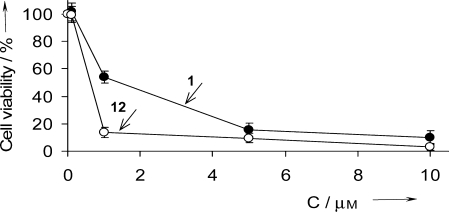
Effects of **1** and **12** on the viability of KB-3-1 cells. Cells were incubated for 24 h in the presence of **1** and **12** (0.1, 5 and 10 μm). Cell viability was measured by MTT assay as described in the Experimental Section. The results are expressed as percentages of viable cells observed after treatment with compounds relative to control cells (100 %) incubated in the presence of DMSO (0.1 % (*v*/*v*)). The data were obtained from three separate experiments in triplicate.

Glycyrrhetinic acid, **7**, **8**, **9**, **10** and **12** were tested; the other derivatives were found to be insoluble in dimethylsulfoxide (DMSO). Cells were exposed to the compounds for 24 h and then assayed for growth by the MTT method. The cells were also incubated in the presence of CDDO-Me **1**, whose ability to inhibit cancer-cell growth was established earlier (reviewed by Liby et al.).[19] Figure 1 shows the dose–response curves for **1** and **12** with KB-3-1 cells, and the IC_50_ values for all the tested compounds (for the inhibition of KB-3-1 cell growth) are presented in Table 1. Compound **12** displayed the highest activity. The in vitro IC_50_ values (the concentrations required for 50 % growth inhibition) were 0.3 and 1.2 μm for **12** and **1**, respectively (Table 1). IC_50_ values for the other glycyrrhetinic acid derivatives were greater than 10 μm.

**Table 1:** IC_50_ values of glycyrrhetinic acid, its derivatives **1**, **7**, **8**, **9**, **10**, **12**.[a]

Compounds	IC_50_ [μm]	Compounds	IC_50_ [μm]
glycyrrhetinic	>10	**8**	>10
acid		**9**	>10
**1**	1.2±0.16	**10**	>10
**7**	>10	**12**	0.3±0.08

[a] IC_50_ was defined as the compound concentration that resulted in 50 % KB-3-1 cell survival as measured by the MTT assay (see Experimental Section). Incubation time: 24 h.

**Figure 2:**
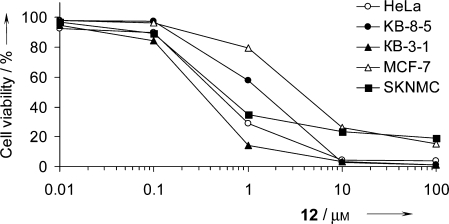
Dose–response curves for **12** with different human cancer cell lines. Cells were incubated for 24 h with increasing concentrations of **12** (0.1 to 100 μm). Cell viability was measured by the MTT assay as described in the Experimental Section. The results are expressed as percentages of viable cells observed after treatment, relative to control cells (100 %) incubated in the presence of DMSO (0.1 % (*v*/*v*)). Data were obtained from three separate experiments in triplicate.

We compared the effects of **12** and **1** on the growth of different human cancer cell-lines: KB-3-1 epidermoid carcinoma cells, KB-8-5 multidrug-resistant cancer cells (a derivative of KB-3-1), HeLa cervical epithelioid carcinoma cells, MCF-7 breast adenocarcinoma cells, and SKNMC neuroblastoma cells. The dose–response curves for **12** with the different cell lines are displayed in Figure 2. Compound **12** induces concentration-dependent cell death in all cell lines tested. IC_50_ values for **12** and **1** are displayed in Table 2. The IC_50_ values for compounds were similar for all cell lines, with the exception of MCF-7, for which IC_50_ was more that ten times higher than for KB-3-1 (5 μm vs 0.3 μm for **12**). IC_50_ values for **12** were lower than for **1** for all tested cell lines.

**Table 2:** IC_50_ values for **12** and oleanolic acid derivative **1** with human cancer cell lines.[a]

Cell line	**12**	**1**
KB-3-1	0.3±0.08	1.2±0.16
KB-8-5	1.2±0.12	3.1±0.29
HeLa	1.3±0.28	2.8±0.37
MCF-7	5±0.34	>10
SK-*N*-MC	0.8±0.1	4.9±0.6

[a] IC_50_ was defined as the compound concentration that resulted in 50 % cell survival as measured by the MTT assay (see Experimental Section). Incubation time: 24 h.

One of the reasons for the failure of chemotherapy-based treatment is multidrug resistance (MDR). We tested the ability of **12** to suppress the growth of multidrug-resistant KB-8-5 cells. This cell line is characterized by overexpression of the *MDR1* gene, which encodes P-glycoprotein, an ATP-dependent membrane pump that efficiently decreases the intracellular concentrations of various compounds. Treatment of KB-8-5 cells with **12** significantly decreased the number of living cells (Figure 2); the IC_50_ value for this cell line was only four times higher than the IC_50_ for the drug-sensitive KB-3-1 (0.3 μm for KB-3-1 vs 1.2 μm for KB-8-5). Thus, this glycyrrhetinic acid derivative is not targeted at P-glycoprotein, and might be efficient against tumors exhibiting the P-glycoprotein-dependent MDR phenotype.

**Figure 3:**
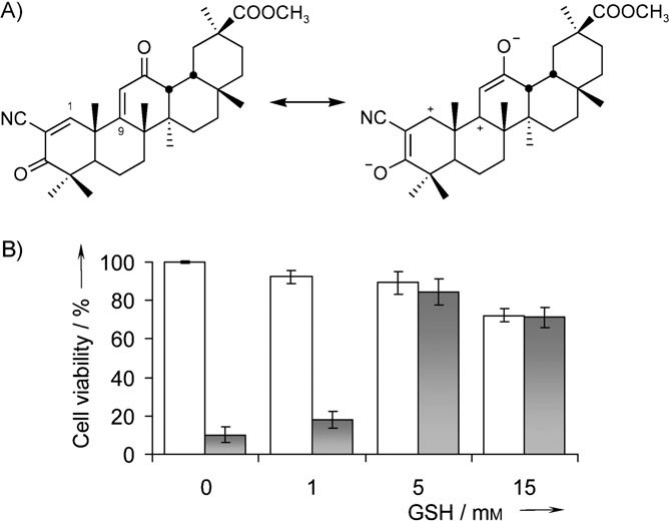
GSH treatment abrogates the cytotoxicity of **12** in KB-3-1 cells. A) Activation of the Michael acceptor sites at positions 1 and 9 of compound **12.** B) Effect of GSH on viability of KB-3-1 cells. Cells were treated for 24 h with the indicated concentrations of either GSH alone (□), or in combination with 1 μm **12** (▪). Cell viability was measured by the MTT assay as described in the Experimental Section. The results are expressed as percentages of viable cells observed after treatment, relative to control cells (100 %) incubated in the presence of DMSO (0.1 % (*v*/*v*)). The data were obtained from three separate experiments done in triplicate.

*The effect of antioxidants on the cytotoxicity of **12**:* Similarly to CDDO and many other synthetic triterpenoids, **12** has potential electrophilic Michael acceptor sites at positions 1 and 9 of the triterpenoid nucleus (Figure 3A). It is known that the presence of Michael acceptor groups at specific positions is essential for inhibition of proliferation, promotion of differentiation, and induction of apoptosis in various cell lines. This arises from the ability of Michael electrophiles to target specific nucleophiles, and to affect selective biological functions.[20–22] The involvement of the Michael electrophiles in a particular biological process can be proved by inhibition of their activity with antioxidants, for example glutathione (GSH).

We investigated whether reducing the nucleophilic agents would abrogate the cytotoxicity of **12**. Cells were incubated in the presence of GSH (1, 5, 15 or 45 μm), either alone or in combination with **12** (1 μm; Figure 3B). Incubation of cells in the presence of **12** with of GSH (5 or 15 μm) decreased the cytotoxicity of **12**. The IC_50_ value in the presence of GSH was 3 μm, but only 0.3 μm for **12** alone. Higher concentrations of GSH were toxic: incubation in the presence of 45 μm glutathione led to 95 % cell death (data not shown). It should be noted that incubation with ascorbic acid did not decrease the cytotoxicity of compound **12** (not shown). Thus, we demonstrated that **12** displays biologically active Michael acceptors.

*Cell-cycle arrest:* Flow cytometry was employed to determine whether **12** caused stage-specific inhibition of the cell cycle (Table 3). After 18 h incubation in the absence of **12,** the number of cells with sub-G_1_ (apoptotic) peak was insignificant. An increase in the concentration of **12** (0.3 to 1 μm ) yielded a corresponding increase in the population of cells in sub-G_1_ (19.2 to 51.8 % ; values relative to the control) in a concentration-dependent manner (*n*=3; *p*<0.05). The increase in the population of cells in the sub-G_1_ phase was accompanied by a decrease of cells in the G_1_ and S phases (Table 3). It has been reported that cells with these features are those dying of apoptosis.[23] The number of cells in the G2-M phase remained constant.

**Table 3:** Effect of **12** on the cell cycle of KB-3-1 cells.[a]

Conc. **12**	Cells in each phase of the cell cycle [%]			
[μm]	Sub-G1	G1	S	G2/M
0	1.7	50.6	22.9	24.8
0.3	19.2	36.5	14.7	29.6
1.0	51.8	11.8	8.3	28.1

[a] KB-3-1 human epidermoid cells were seeded into six-well plates to ensure that they had not reached confluency. After 24 h they were incubated either in the absence (control) or presence of 0.3 or 1 μm **12**. After 18 h the percentage of cells in each phase of the cell cycle was determined by flow cytometry as described in the Experimental Section. Data were obtained from at least three separate experiments in duplicate.

**Figure 4:**
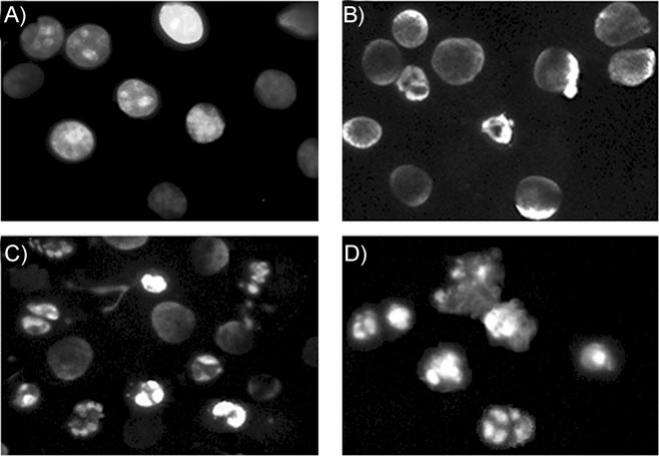
Nuclear damage in KB-3-1 cells by treatment with 1 μm **12.** Photos were taken by fluorescence microscopy after nuclei staining with Hoechst 33258. The figures show the microscopic morphology of the cells incubated in the presence of A) DMSO (0.1 % (*v*/*v*), control), and in the presence of **12** for B) 6, C) 18, and D) 24 h.

*Morphological observation of nuclear change:* There are several morphological characteristics for apoptotic cells, such as cell shrinkage, nuclear fragmentation and chromatin condensation. To examine cell death due to exposure to **12,** we investigated the nuclear morphological changes in KB-3-1 cells treated with 1 μm **12** for 6, 18 and 24 h (Figure 4). Nuclear staining with Hoechst 33258 demonstrated that control KB-3-1 cells had regular and round-shaped nuclei (Figure 4A). In contrast, condensation and fragmentation of nuclei, characteristic of apoptotic cells, were observed in cells treated with **12**. After 6 h exposure, patches of localized partially condensed chromatin were found on the inner face of the nuclear membrane (Figure 4B), while the nuclei appeared slightly deformed. The number of such nuclei dramatically increased up to 18 h (Figure 4C). Incubation of cells for 24 h led to severe damage to the vast majority of nuclei, with the formation of apoptotic bodies (Figure 4D).

**Figure 5:**
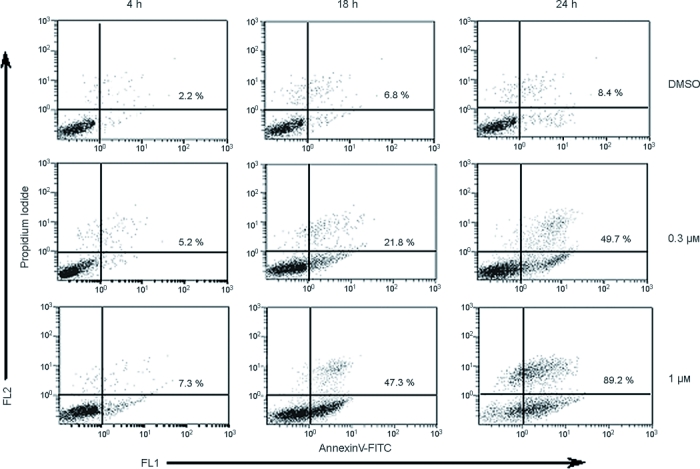
Quantification of apoptosis by annexin V binding to KB-3-1 cells. Cells were incubated in the presence of 0.3 or 1 μm **12,** or in the presence of 0.1 % (*v*/*v*) DMSO for the indicated times. Annexin V binding was carried out with the Annexin V–FITC detection kit as described in the Experimental Section. Annexin V/PI staining was analyzed by flow cytometry. The lower-right quadrant of each cytometry scattergram shows the annexinV^+^PI^−^ cells; the upper-right quadrant shows the annexinV^+^PI^+^ cells. The results are representive of one of three independent experiments.

*Quantification of apoptosis by annexin V binding and flow cytometry:* Increases in morphologically changed cells, and in the number of cells in the sub-G0/G1 phase, are usually associated with apoptosis. We examined whether cell death was apoptotic when induced by the glycyrrhetinic acid derivatives by using annexin V and propidium iodide analysis (Figure 5). KB-3-1 cells were exposed to **12**, then subjected to flow cytometric analysis. Annexin V binds phosphatidylserine residues, which are asymmetrically distributed toward the inner plasma membrane, and migrate to the outer plasma membrane during apoptosis.[24] The data show that **12** induced apoptotic cell death in 50 % of KB-3-1 cells at concentrations equal to the IC_50_ values. The number of apoptotic cells increased with the time of incubation, and with increasing compound concentration. 89.2 % of KB-3-1 cells were detected as apoptotic following 24 h of incubation in the presence of 1 μm **12**, so **12** induces dose- and time-depended apoptotic cell death. Taken together, these data indicate that the decrease in viability of cancer cells exposed to the novel glycyrrhetinic acid derivatives occurred by apoptosis, and that **12** had the greatest potency.

**Figure 6:**
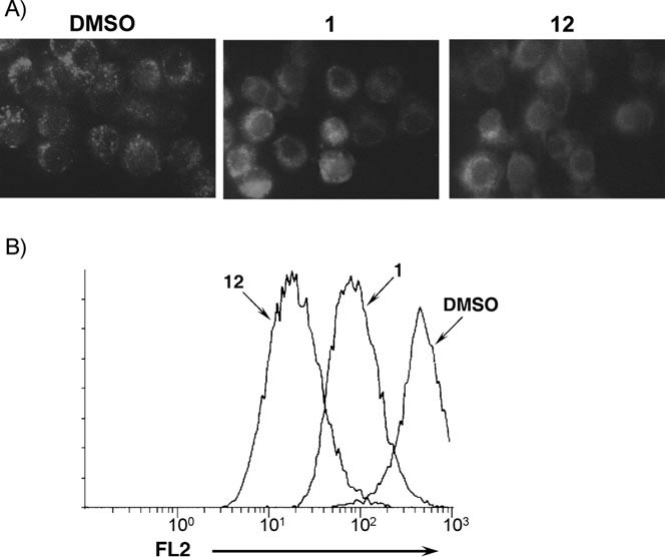
Analysis of mitochondrial transmembrane potential of KB-3-1 cells treated with **1** and **12** by A) fluorescence microscopy, and B) flow cytometry, following JC-1 staining. Cells were treated for 6 h with **1** or **12** (1 μm), or DMSO (0.1 % (*v*/*v*)). In normal cells dye accumulated in mitochondria (red fluorescence). In cells with altered mitochondrial potential dye remained as monomers in the cytoplasm (green fluorescence). FL2=value of fluorescence measured on channel 2 of the cytofluorometer.

*Dissipation of the mitochondrial transmembrane potential:* We investigated whether **12** utilizes the mitochondrial “intrinsic” pathway in the apoptotic death of KB-3-1 cells, as the pivotal role of mitochondria in the triggering of apoptosis is well established. We evaluated the mitochondrial transmembrane potential (Δ*ψ*_m_) in KB-3-1 cells exposed to **12,** and compared this to that for **1**, whose ability to decrease Δ*ψ*_m_ has been documented.[10, 19, 25–29] Changes in Δ*ψ*_m_ were evaluated by cytofluorometric analysis. Cells were stained with the mitochondria-specific cationic dye JC-1 (5,5′,6,6′-tetrachloro-1,1′,3,3′-tetraethyl benzimidazole carbocyanine iodide), which accumulates in the transmembrane region of polarized mitochondria where it forms “J-aggregates”. These emit orange fluorescence that can be recorded on channel 2 of a cytofluorometer, or visualized via a red filter on a fluorescence microscope. A decrease in Δ*ψ*_m_ results in a decrease in J-aggregates and increase in JC-1 monomers, which emit a greenish-yellow fluorescence. The cytometric analysis of KB-3-1 cells stained with JC-1 is shown in Figure 6B. In the control cells (incubated in the presence of 0.1 % DMSO) the majority of cells showed a high emission of fluorescence in both channels, because of the equilibrium between J-aggregates and monomers. The exposure of KB-3-1 cells to **12** leads to a significant decrease in fluorescence compared to the control (0.1 % DMSO). In fluorescent microscopy (Figure 6A), one can see that most of the cells turn green. After incubation in the presence of **1,** cells can be seen to be somewhere between the control and **12**-treated cells, both in the fluorescent micrograph and in the flow-cytometry histogram. One can conclude that **12** causes a dramatic dissipation of mitochondrial potential, and that this effect, consistent with the results of the MTT assay, is more pronounced than that for **1**.

**Figure 7:**
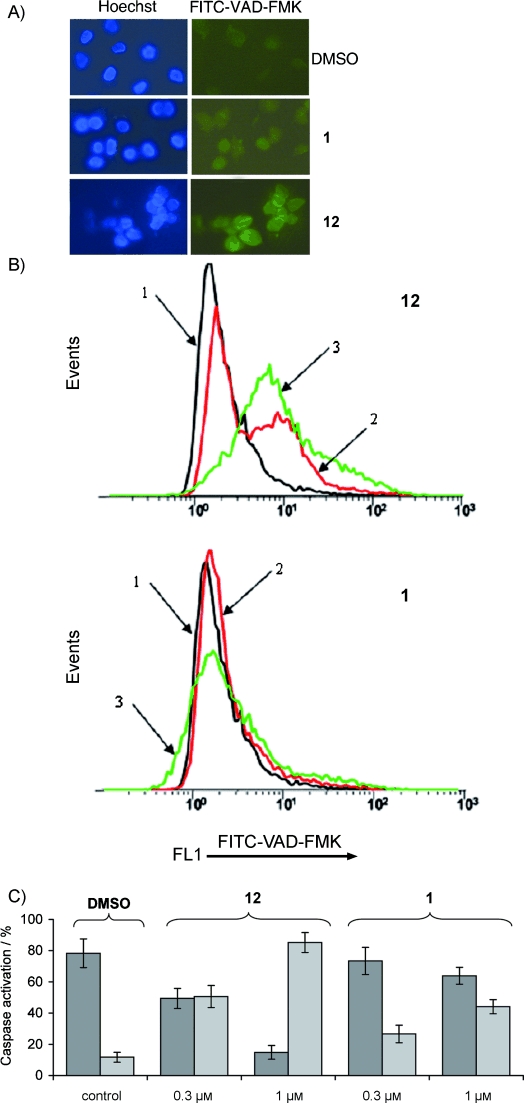
Induction of caspase activation in human cancer cells. A) Fluorescence microscopy of FITC-VAD-FMK in living KB-3-1 cells incubated for 18 h with 0.3 μm **1** or **12**. Cells were counter-stained with Hoechst dye. B) Flow cytometry analysis of the activation of proteins of the caspase family. Cytograms of FITC-VAD-FMK in living cells incubated with **1** or **12**. KB-3-1 cells without treatment (1), treated with 0.3 μm (2) and 1 μm (3) of the indicated compounds (18 h incubation). C) Percentages of cells undergoing apoptosis (staining positive for FITC-VAD-FMK). Percentage of cells with non-activated (▪) and activated (▪) caspases after treatment with the indicated concentrations of **1**, **12** or DMSO (0.1 % (*v*/*v*), control). Error bars indicate standard deviation.

*Activation of the caspase cascade in apoptosis induced by glycyrrhetinic acid derivatives:* To determine whether activation of the caspase cascade is involved in **12**-induced apoptosis, we used the fluorescein isothiocyanate (FITC)-labeled pan-caspase inhibitor FITC-VAD-FMK (FITC-valyl-alanyl-aspartyl-[O-methyl]-fluoromethylketone). The conjugated compound is cell-permeable and binds irreversibly to activated caspase molecules, and thus serves as an in situ marker for apoptosis.[30] We compared the abilities of **12** and **1** to activate caspase (Figure 7). In control cells (18 h incubation in the presence of 0.1 % DMSO) only a faint green signal was seen: this equates to 9 % of cells with activated caspase (Figure 7A and C). With the addition of **12** (0.3 and 1 μm), the number of cells with activated caspase increased (51 and 85 %, respectively; Figure 7C), and green fluorescence was observed in the fluorescence microscope. Similar assays with **1** yielded data that lay between those for the control and **12**, as had been the case for mitochondrial transmembrane potential dissipation.

The results provide evidence that the most-active glycyrrhetinic-acid derivative **12** induces caspase-dependent apoptosis in cancer cells. Caspase involvement in cell death is suggested also by the higher IC_50_ for MCF-7 cells (Table 2)—cells that are known to be caspase-3-deficient.[31]

## Conclusions

In this report we describe the synthesis of the new glycyrrhetinic acid derivative methyl 2-cyano-3,12-dioxo-18βH-olean-9(11),1(2)-dien-30-oate (**12**), obtained by the direct modification of the A and C rings of glycyrrhetinic acid. We provide a detailed description of synthesis and physicochemical characteristics of the end product, **12**, and of the intermediate compounds. The modifications converted the well-known triterpenoid (exhibiting weak antitumor activity) to derivative **12,** which displays high antiproliferative activity toward cancer cells. The intermediate products **7**–**10** did not display this activity.

We have shown that human epidermoid cancer cells are sensitive to **12**, as are other tumor cell types, including cells exhibiting the multidrug-resistant phenotype. Compound **12** displays potent single-agent activity, at micromolar concentrations, against different human cancer cells in culture. The mechanism of action (MOA) of triterpenoids on cancer cells is not fully understood. Different mechanisms have been proposed for the cytotoxic activity of synthetic triterpenoids in various types of cancer and leukemia cells; this suggests that cellular context is important. Several studies point to an MOA dependent on the extrinsic apoptotic pathway (DR4/DR5/caspase-8 activation),[20, 32] whereas other studies point to involvement of the intrinsic apoptotic pathway.[10, 33, 34] Our studies imply that the apoptotic MOA of **12** includes components of intrinsic pathways in epidermoid cancer cells.

We have compared the ability of **12** to cause cancer-cell death with that of CDDO-Me, a well known compound that is currently in late-stage clinical trials for the treatment of chronic kidney disease in type 2 diabetes mellitus patients. The antiproliferative activity of **12** exceeds that of CDDO-Me: the IC_50_ value was lower for all tested cell lines, and **12** caused a dramatic dissipation of mitochondrial potential and caspase-cascade activation; these effects were more pronounced for **12** than for CDDO-Me. Moreover, we can suppose that commercial synthesis of **12** will be more amenable than that of CDDO-Me because 18βH-glycyrrhetinic acid, the starting material for the synthesis of **12**, is more readily available than oleanolic acid. Indeed, the level of oleanolic acid in olive leaves ranges from 0.71 to 3.5 %,[35–37] and from 0.8 to 4.3 % in *Ligustrum* fruit;[38] the level of glycyrrhizin can reach 25–30 % in *Glycyrrhiza glabra* root, and 90 % in triterpene extracts of licorice root.[39–41]

## Experimental Section

**Reagents:** CDDO-Me **1** was synthesized from oleanolic acid according to a previously described method[5] with 10 % yield (NMR ^1^H and ^13^C data).

18βH-Glycyrrhetinic acid acetate **2**, obtained from a licorice extract, was used as starting material (purity∼94 %).[39]

**General experimental procedures:** Melting points were determined on a Hoover melting point apparatus and were uncorrected. The elemental composition of the products was determined from high-resolution mass spectra recorded on a DFS (Double Focusing Sector) Thermo Electron Corporation instrument. ^1^H and ^13^C NMR spectra were measured from CDCl_3_ solutions on Bruker spectrometers: AM-400 (400.13mhz for ^1^H, 100.61mhz for ^13^C) and DRX-500 (500.13mhz for ^1^H, 125.76mhz for ^13^C). Chloroform was used as the internal standard (*δ*_H_ 7.24 ppm, *δ*_C_ 76.90 ppm). The structure of the compounds was determined by NMR from proton spin–spin coupling constants in ^1^H,^1^H double-resonance spectra, and by analyzing ^13^C NMR proton-selective and off-resonance saturation spectra, 2D ^13^C,^1^H correlated spectroscopy on CH constants (COSY, ^1^*J*_C,H_=135 Hz; and COLOC, ^2, 3^*J*_C,H_=10 Hz, correspondingly), and 1D ^13^C,^1^H long-range *J* modulation difference (LRJMD, *J*_C,H_=10 Hz). Flash column chromatography was performed with silica gel (Merck, 60–200 mesh) and neutral alumina (Chemapol, 40–250 mesh).

**Methyl 18βH-Glycyrrhetinate acetate (3):**[42] A solution of diazomethane in ether was added dropwise at 0°C to a stirred suspension of **2** (10 g, 19.0 mmol) in methanol (200 mL) until the originally colorless mixture turned yellow. The resulting mixture was allowed to stand at room temperature overnight. The solvent was removed and the product was purified by crystallization (chloroform/methanol; yield=9.1 g, 89 %). M.p. 303–304°C; ^1^H NMR (CDCl_3_): *δ*=0.76 (dd, ^3^*J*(H^5a^,H^6a^)=12.5, ^3^*J*(H^5a^,H^6e^)=1.5 Hz; H^5a^), 0.76 (s, 3 H; C28-H_3_), 0.84 (s, 6 H; C23-H_3_, C24-H_3_), 0.97 (dm, ^2^*J*(H^16e^,H^16a^)=13.8 Hz; H^16e^), 1.01 (ddd, ^2^*J*(H^1a^,H^1e^)=13.5, ^3^*J*(H^1a^,H^2a^)=13.5, ^3^*J*(H^1a^,H^2e^)=3.7 Hz; H^1a^), 1.08 (s, 3 H; C26-H_3_), 1.10 (s, 3 H; C29-H_3_), 1.12 (s, 3 H; C25-H_3_), 1.14 (dm, ^2^*J*(H^15e^_,_H^15a^)=13.8 Hz; H^15e^), 1.23–1.39 (m, 4 H; H^7^, H^21a^, 2 H^22^), 1.32 (s, 3 H; C27-H_3_), 1.41 (dddd, ^2^*J*(H^6a^_,_H^6e^)=13.5, ^3^*J*(H^6a^_,_H^7a^)=13.5, ^3^*J*(H^6a^_,_H^5a^)=12.0, ^3^*J*(H^6a^,H^7e^)=3.2 Hz; H^6a^), 1.51–1.66 (m, 3 H; H^6e^, H^2e^, H^7′^), 1.57 (dd, ^2^*J*(H^19a^,H^19e^)=13.5, ^3^*J*(H^19a^,H^18a^)=13.5 Hz; H^19a^), 1.66 (dddd, ^2^*J*(H^2a^,H^2e^)=13.5, ^3^*J*(H^2a^,H^1a^)=13.5, ^3^*J*(H^2a^,H^3a^)=11.7, ^3^*J*(H^2a^,H^1e^)=3.7 Hz; H^2a^), 1.78 (ddd, ^2^*J*(H^15a^,H^15e^)=13.8, ^3^*J*(H^15a^,H^16a^)=13.8, ^3^*J*(H^15a^_,_H^16e^)=4.5 Hz; H^15a^), 1.88 (ddd, ^2^*J*(H^19e^,H^19a^)=13.5, ^3^*J*(H^19e^,H^18a^)=4.2, ^4^*J*(H^19e^,H^21e^)=2.7 Hz; H^19e^), 1.95 (dm ^2^*J*(H^21e^,H^21a^)=10 Hz; H^21e^), 1.98 (ddd, ^2^*J*(H^16a^,H^16e^)=13.8, ^3^*J*(H^16a^,H^15a^)=13.8, ^3^*J*(H^16a^,H^15e^)=4.8 Hz; H^16a^), 2.00 (s, 3 H; C33-H_3_), 2.04 (dd, ^3^*J*(H^18a^,H^19a^)=13.5, ^3^*J*(H^18a^,H^19e^)=4.2 Hz; H^18a^), 2.32 (s, 1 H; H^9a^), 2.76 (ddd ^2^*J*(H^1e^,H^1a^)=13.5, ^3^*J*(H^1e^,H^2a^)=3.7, ^3^*J*(H^1e^,H^2e^)=3.0 Hz; H^1e^), 3.64 (s, 3 H; OC31-H_3_), 4.47 (dd, ^3^*J*(H^3a^,H^2a^)=11.7, ^3^*J*(H^3a^,H^2e^)=4.7 Hz; H^3a^), 5.62 (s, 1 H; H^12^); ^13^C NMR (CDCl_3_): *δ*=38.63 (t, C1), 23.41 (t, C2), 80.45 (d, C3), 37.88 (s, C4), 54.88 (d, C5), 17.22 (t, C6), 32.55 (t, C7), 43.03 (s, C8), 61.56 (d, C9), 36.78 (s, C10), 199.85 (s, C11), 128.34 (d, C12), 169.01 (s, C13), 45.23 (s, C14), 26.31 (t, C15), 26.26 (t, C16), 31.67 (s, C17), 48.25 (d, C18), 40.93 (t, C19), 43.87 (s, C20), 30.98 (t, C21), 37.59 (t, C22), 27.89 (q, C23), 16.52 (q, C24), 16.24 (q, C25), 18.52 (q, C26), 23.17 (q, C27), 28.36 (q, C28), 28.15 (q, C29), 176.73 (s, C30), 51.58 (q, C31), 170.77 (s, C32), 21.13 (q, C33); HRMS: *m/z* calcd for C_33_H_50_O_5_: 526.7471; found: 526.3658.

**Methyl 3 β-Acetoxy-18 βH-olean-12-en-30-oate (4):**[43] A solution of conc. hydrochloric acid (50 mL) was added dropwise at 10°C to a stirred suspension of **3** (9.1 g, 17.3 mmol) and zinc powder (18.2 g, 280 mmol) in dioxane (300 mL) over 2 h. The reaction mixture was stirred for a further 3 h at 5–10°C, concentrated in a vacuum, diluted with water (1 L), and filtered. The solid was dried and subjected to flash column chromatography (silica gel; benzene followed by chloroform) to give crude **4** (yield=6.8 g, 77 %). This material was used for the next reaction without further purification. An analytically pure sample was obtained by recrystallization from a mixture chloroform/methanol. M.p. 265–267°C; ^1^H NMR (CDCl_3_): *δ*=0.74 (s, 3 H; C28-H_3_), 0.81 (dd, ^3^*J*(H^5a^,H^6a^)=12.0, ^3^*J*(H^5a^,H^6e^)=1.6 Hz; H^5a^), 0.82–0.86 (m, H^16e^), 0.83 (s, 3 H; C24-H_3_), 0.84 (s, 3 H; C23-H_3_), 0.93 (s, 6 H; C25-H_3_, C26-H_3_), 0.94 (dm ^2^*J*(H^15e^,H^15a^)=13.5 Hz; H^15e^), 1.02 (m; H^1^), 1.09 (s, 3 H; C29-H_3_), 1.10 (s, 3 H; C27-H_3_), 1.17–1.35 (m, 4 H; H^7^, H^21^, 2 H^22^), 1.39 (m, H^6a^), 1.44–1.64 (m, 7 H; H^1′^, 2 H^2^, H^6e^, H^7′^, H^9a^, H^19^), 1.73 (ddd, ^2^*J*(H^15a^,H^15e^)=13.5, ^3^*J*(H^15a^,H^16a^)=13.5, ^3^*J*(H^15a^,H^16e^) 4.6 Hz; H^15a^), 1.79–1.93 (m, 5 H; 2 H^11^, H^18^, H^19′^, H^21′^), 1.92 (m; H^16a^), 2.01 (s, 3 H; C33-H_3_), 3.64 (s, 3 H; OC31-H_3_), 4.47 (dd, ^3^*J*(H^3a^,H^2a^)=10.0, ^3^*J*(H^3a^,H^2e^)=6.0 Hz; H^3a^), 5.23 (t, ^2^*J*(H^12^,H^11^)=3.6 Hz; H^12^); ^13^C NMR (CDCl_3_): *δ*=38.13 (t, C1), 23.42 (t, C2), 80.74 (d, C3), 37.56 (s, C4), 55.13 (d, C5), 18.11 (t, C6), 32.46 (t, C7), 39.65 (s, C8), 47.42 (d, C9), 36.70 (s, C10), 23.34 (t, C11), 122.34 (d, C12), 144.23 (s, C13), 41.38 (s, C14), 25.99 (t; C15), 26.82 (t, C16), 31.79 (s, C17), 48.05 (d, C18), 42.68 (t, C19), 44.12 (s, C20), 31.15 (t, C21), 38.25 (t, C22), 27.89 (q, C23), 16.54 (q, C24), 15.41 (q, C25), 16.64 (q, C26), 25.77 (q, C27), 28.03 (q, C28), 28.38 (q, C29), 177.46 (s, C30), 51.35 (q, C31), 170.77 (s, C32), 21.13 (q, C33); HRMS: *m/z* calcd for C_33_H_52_O_4_: 512.7636; found: 512.3866.

**Methyl 3β-acetoxy-12-oxo-18βH-olean-30-oate (5):** A mixture of hydrogen peroxide (∼30 %, 25 mL) and acetic acid (25 mL) was added dropwise at 80°C to a stirred suspension of **4** (3.0 g, 5.7 mmol) in acetic acid (100 mL) over 1 h. The reaction mixture was stirred for a further 30 min at 80°C, cooled to room temperature, and diluted with water (500 mL). The solid was filtered, washed with water, and dried to give crude **5** (yield=6.8 g, 96 %). This material was used for the next reaction without further purification. An analytically pure sample was obtained by recrystallization from a mixture chloroform/methanol. M.p. 296–299°C; ^1^H NMR (CDCl_3_): *δ*=0.79–0.88 (m, 2 H; H^5a^, H^16e^), 0.82 (s, 3 H; C28-H_3_), 0.83 (s, 3 H; C24-H_3_), 0.84 (s, 3 H; C23-H_3_), 0.86 (s, 3 H; C25-H_3_), 0.90 (s, 3 H; C27-H_3_), 0.92–1.01 (m, 2 H; H^1^, H^15e^), 1.09 (s, 3 H; C29-H_3_), 1.10 (s, 3 H; C26-H_3_), 1.19 (dd, ^2^*J*(H^19a^,H^19e^)=13.4, ^3^*J*(H^19a^,H^18^)=13.4 Hz; H^19a^), 1.18–1.27 (m, 2 H; H^21a^, H^22e^), 1.31–1.48 (m, 4 H; 2 H^7^, H^22a^, H^6^), 1.49–1.64 (m, 4 H; H^1′^, 2 H^2^, H^6′^), 1.64 (dd ^3^*J*(H^9a^,H^11a^)=13.0, ^3^*J*(H^9a^,H^11e^)=5.1 Hz; H^9a^), 1.74 (m, H^18^), 1.76 (m, H^15a^), 1.85 (ddd, ^2^*J*(H^16a^,H^16e^)=13.2, ^3^*J*(H^16a^,H^15a^)=13.2, ^3^*J*(H^16a^,H^15e^)=4.2 Hz; H^16a^), 1.91 (dm, ^2^*J*(H^21e^,H^21a^)=13.2 Hz; H^21e^), 2.00 (s, 3 H; C33-H_3_), 2.12 (dd, ^2^*J*(H^11a^,H^11e^)=17.0, ^3^*J*(H^11a^,H^9a^)=13.0 Hz; H^11a^), 2.23 (dd, ^2^*J*(H^11e^,H^11a^)=17.0, ^3^*J*(H^11e^,H^9a^)=5.1 Hz; H^11e^), 2.54 (ddd, ^2^*J*(H^19e^,H^19a^)=13.4, ^3^*J*(H^19e^,H^18^)=3.4, ^4^*J*(H^19e^,H^21e^)=2.8 Hz; H^19e^), 2.72 (d, ^3^*J*(H^13^,H^18^)=4.4 Hz; H^13^), 3.68 (s, 3 H; OC31-H_3_), 4.44 (dd, ^3^*J*(H^3a^,H^2a^)=11.4, ^3^*J*(H^3a^,H^2e^)=4.8 Hz; H^3a^); ^13^C NMR (CDCl_3_): *δ*=37.50 (t, C1), 23.26 (t, C2), 80.28 (d, C3), 37.60 (s, C4), 55.04 (d, C5), 18.05 (t, C6), 31.63 (t, C7), 41.41 (s, C8), 49.32 (d, C9), 36.67 (s, C10), 38.28 (t, C11), 211.91 (s, C12), 50.08 (d, C13), 41.90 (s, C14), 25.84 (t, C15), 26.29 (t, C16), 32.01 (s, C17), 38.41 (d, C18), 34.05 (t, C19), 43.97 (s, C20), 31.19 (t, C21), 38.33 (t, C22), 27.75 (q, C23), 16.30 (q, C24), 15.16 (q, C25), 15.95 (q, C26), 20.78 (q, C27), 26.83 (q, C28), 28.59 (q, C29), 177.44 (s, C30), 51.36 (q, C31), 170.70 (s, C32), 21.09 (q, C33); HRMS: *m/z* calcd for C_33_H_52_O_5_: 528.7630; found: 528.3815.

**Methyl 3β-acetoxy-12-oxo-18β*H*-olean-9(11)-en-30-oate (6):** Compound **6** was synthesized according to a known method.[44] Briefly, a solution of bromine (0.5 mL, 9.8 mmol) in glacial acetic acid (50 mL) was added dropwise at 80°C to a stirred solution of **5** (4.0 g, 7.6 mmol) in glacial acetic acid (150 mL) over 1 h. The reaction mixture was stirred for a further 1 hour at 80°C, cooled to room temperature, diluted with water (1.5 L) and filtered. The solid was washed with water, dried, and subjected to flash column chromatography (silica gel, chloroform) to give a solid **6** (yield=3.0 g, 75 %). This material was used for the next reaction without further purification. An analytically pure sample was obtained by recrystallization from a mixture chloroform/methanol. M.p. 298°C; ^1^H NMR (CDCl_3_): *δ*=0.84 (m; H^16e^), 0.85 (s, 3 H; C24-H_3_), 0.86 (s, 6 H; C23-H_3_, C28-H_3_), 0.91 (s, 3 H; C27-H_3_), 0.94 (dm, ^3^*J*(H^5a^,H^6a^)=10 Hz; H^5a^), 1.01 (dm, ^2^*J*(H^15e^,H^15a^)=13.2 Hz; H^15e^), 1.07 (s, 3 H; C29-H_3_), 1.16 (s, 3 H; C25-H_3_), 1.20 (dd, ^2^*J*(H^19a^,H^19e^)=13.3, ^3^*J*(H^19a^,H^18^)=13.3 Hz; H^19a^), 1.16–1.27 (m, 2 H; H^21a^, H^22e^), 1.32 (s, 3 H; C26-H_3_), 1.35–1.44 (m, 2 H; H^1a^, H^7e^), 1.46 (ddd, ^2^*J*(H^22a^,H^22e^)=14.0, ^3^*J*(H^22a^,H^21a^)=14.0, ^3^*J*(H^22a^,H^21e^)=4.2 Hz; H^22a^), 1.55–1.73 (m, 5 H; 2 H^6^, H^7a^, 2 H^2^), 1.77 (m; H^15a^), 1.83 (m; H^16a^), 1.90 (m, 2 H; H^1e^, H^21e^), 1.93 (dm, ^3^*J*(H^18^,H^19a^)=13.3; H^*18*^), 2.00 (s, 3 H; C33-H_3_), 2.16 (ddd, ^2^*J*(H^19e^,H^19a^)=13.3, ^3^*J*(H^19e^,H^18^)=3.4, ^4^*J*(H^19e^,H^21e^)=2.8 Hz; H^19e^), 2.92 (d, ^3^*J*(H^13^,H^18^)=4.7 Hz; H^13^), 3.69 (s, 3 H; OC31-H_3_), 4.43 (dd, ^3^*J*(H^3a^,H^2a^)=11.7, ^3^*J*(H^3a^,H^2e^)=4.5 Hz; H^3a^), 5.72 (s; H^11^); ^13^C NMR (CDCl_3_): *δ*=35.86 (t, C1), 23.66 (t, C2), 79.49 (d, C3), 37.95 (s, C4), 50.08 (d, C5), 17.67 (t, C6), 32.66 (t, C7), 45.29 (s, C8), 177.61 (s, C9), 39.59 (s, C10), 122.89 (d, C11), 201.14 (s, C12), 47.70 (d, C13), 41.61 (s, C14), 26.03 (t, C15), 26.03 (t, C16), 31.87 (t, C17), 37.81 (d, C18), 33.69 (t, C19), 43.88 (s, C20), 31.10 (t, C21), 38.13 (t, C22), 27.77 (q, C23), 16.46 (q, C24), 23.79 (q, C25), 23.85 (q, C26), 21.84 (q, C27), 26.90 (q, C28), 28.45 (q, C29), 177.22 (s, C30), 51.32 (q, C31), 170.59 (s, C32), 21.01 (q, C33); HRMS: *m/z* calcd for C_33_H_50_O_5_: 526.7471; found: 526.3658.

**Methyl 3β-hydroxy-12-oxo-18βH-olean-9(11)-en-30-oate (7):** A mixture of **6** (6.2 g, 11.8 mmol) and KOH (41 g, 732 mmol) in methanol (400 mL) was heated under reflux for 1.5 h. The resulting solution was cooled to room temperature, concentrated in vacuo, and 10 % aqueous hydrochloric acid solution was added. The mixture was extracted with chloroform/ethyl acetate (1:4, 3×75 mL). The combined organic layers were washed with saturated sodium hydrogen carbonate solution (3×50 mL) and brine (3×50 mL), and dried over magnesium sulfate. The solvent was removed to give a solid **7** (yield=5.13 g, 90 %). This material was used for the next reaction without further purification. An analytically pure sample was obtained by recrystallization from a mixture chloroform/methanol. M.p. 202–203°C; ^1^H NMR: *δ*=0.80 (s, 3 H; C24-H_3_), 0.89 (s, 3 H; C28-H_3_), 0.94 (s, 3 H; C27-H_3_), 1.01 (s, 3 H; C23-H_3_), 1.08 (s, 3 H; C29-H_3_), 1.16 (s, 3 H; C25-H_3_), 1.35 (s, 3 H; C26-H_3_), 0.83–0.91 (m, 2 H; H^5a^, H^16e^), 1.04 (dm, ^2^*J*(H^15e^,H^15a^)=12.8 Hz; H^15e^), 1.23 (dd, ^2^*J*(H^19a^,H^19e^)=13.2, ^3^*J*(H^19a^,H^18^)=13.2 Hz; H^19a^), 1.18–1.25 (m; H^21a^), 1.27 (dm, ^2^*J*(H^22e^,H^22a^)=14.0 Hz; H^22e^), 1.31 (m; H^1a^), 1.44 (dm, ^2^*J*(H^7e^,H^7a^)=9.8 Hz; H^7e^), 1.49 (ddd, ^2^*J*(H^22a^,H^22e^)=14.0, ^3^*J*(H^22a^,H^21a^)=14.0, ^3^*J*(H^22a^,H^21e^)=4.2 Hz; H^22a^), 1.55–1.76 (m, 5 H; 2 H^6^, H^7a^, 2 H^2^), 1.80 (m, H^15a^), 1.85 (m; H^16a^), 1.89–2.00 (m, 3 H; H^1e^, H^21e^, H^18^), 2.19 (ddd, ^2^*J*(H19^e^,19^a^)=13.2, ^3^*J*(H^19e^,H^18^)=3.2, ^4^*J*(H^19e^,H^21e^)=2.7 Hz; H^19e^), 2.95 (d, ^3^*J*(H^13^,H^18^) 4.7 Hz; H^13^), 3.18 (dd ^3^*J*(H^3a^,H^2a^)=11.7, ^3^*J*(H^3a^,H^2e^)=4.4 Hz; H^3a^), 3.71 (s, 3 H; OC31-H_3_), 5.76 (s, H^11^); ^13^C NMR (CDCl_3_): *δ*=36.22 (t, C1), 27.38 (t, C2), 77.83 (d, C3), 39.11 (s, C4), 50.06 (d, C5), 17.87 (t, C6), 32.79 (t, C7), 45.39 (s, C8), 178.10 (s, C9), 39.81 (s, C10), 122.85 (d, C11), 201.36 (s, C12), 47.76 (d, C13), 41.70 (s, C14), 26.14 (t, C15), 26.12 (t, C16), 31.95 (s, C17), 37.88 (d, C18), 33.79 (t, C19), 43.95 (s, C20), 31.17 (t, C21), 38.21 (t, C22), 27.96 (q, C23), 15.44 (q, C24), 23.81 (q, C25), 23.87 (q, C26), 21.99 (q, C27), 26.96 (q, C28), 28.52 (q, C29), 177.33 (s, C30), 51.43 (q, C31); HRMS: *m/z* calcd for C_31_H_48_O_4_: 484.7104; found: 484.3553.

**Methyl 3,12-Dioxo-18βH-olean-9(11)-en-30-oate (8):** Jones reagent (5 mL), prepared from Na_2_Cr_2_O_7_**⋅**2 H_2_O in dilute sulfuric acid (33 %)[45] was added dropwise to a solution of **7** (5.13 g) in acetone (500 mL) at 0°C over 30 min till the brown color persisted. The mixture was stirred for further 2.5 h at room temperature, and ethanol (10 mL) was added. The resulting mixture was concentrated in a vacuum (∼100 mL), and water (1 L) was added. The solid was filtered and dried. The ketone **8** was purified by column chromatography (neutral alumina, chloroform) (yield=4.7 g, 94 %). This material was used for the next reaction without further purification. An analytically pure sample was obtained by recrystallization from a mixture chloroform/methanol. M.p. 189–192°C; ^1^H NMR (CDCl_3_): *δ*=0.88 (s, 3 H; C28-H_3_), 0.94 (s, 3 H; C27-H_3_), 1.05 (s, 3 H; C24-H_3_), 1.07 (s, 3 H; C29-H_3_), 1.08 (s, 3 H; C23-H_3_), 1.27 (s, 3 H; C25-H_3_), 1.38 (s, 3 H; C26-H_3_), 0.89 (m, H^16e^), 1.06 (m, H^15e^), 1.21 (dd, ^2^*J*(H^19a^,H^19e^)=13.3, ^3^*J*(H^19a^,H^18^)=13.3 Hz; H^19a^), 1.17–1.29 (m, 2 H; H^21a^, H^22e^), 1.43–1.50 (m, 2 H; H^5a^, H^7^), 1.48 (ddd, ^2^*J*(H^22a^,H^22e^)=14.0, ^3^*J*(H^22a^,H^21a^)=14.0, ^3^*J*(H^22a^,H^21e^)=4.3 Hz; H^22a^), 1.60–1.72 (m, 3 H; 2 H^6^, H^7′^), 1.75 (m, H^1a^), 1.80 (m; H^15a^), 1.85 (m, H^16a^), 1.92 (m, H^*21e*^), 1.96 (ddd, ^3^*J*(H^18^,H^19a^)=13.3, ^3^*J*(H^18^,H^13^)=4.7, ^3^*J*(H^18^,H^19e^)=3.2 Hz; H^18^), 2.16 (m, H^1e^), 2.17 (ddd, ^2^*J*(H^19e^,H^19a^)=13.3, ^3^*J*(H^19e^,H^18^)=3.2, ^4^*J*(H^19e^,H^21e^)=2.8 Hz; H^19e^), 2.44 (ddd, ^2^*J*(H^2e^,H^2a^)=15.8, ^3^*J*(H^2e^,H^1a^)=7.2, ^3^*J*(H^2e^,H^1e^)=3.8 Hz; H^2e^), 2.60 (ddd, ^2^*J*(H^2a^,H^2e^)=15.8, ^3^*J*(H^2a^,H^1a^)=11.5, ^3^*J*(H^2a^,H^1e^)=7.3 Hz; H^2a^), 2.98 (d, ^3^*J*(H^13^,H^18^)=4.7 Hz; H^13^), 3.70 (s, OC31-H_3_), 5.78 C(H^11^); ^13^C NMR (CDCl_3_): *δ*=36.80 (t, C1), 33.97 (t, C2), 215.57 (s, C3), 47.35 (s, C4), 50.73 (d, C5), 18.97 (t, C6), 31.90 (t, C7), 45.46 (s, C8), 176.36 (s, C9), 39.23 (s, C10), 124.10 (d, C11), 200.78 (s, C12), 47.86 (d, C13), 41.78 (s, C14), 26.13 (t, C15), 26.11 (t, C16), 31.90 (s, C17), 37.82 (d, C18), 33.76 (t, C19), 43.90 (s, C20), 31.12 (t, C21), 38.14 (t, C22), 26.10 (q, C23), 21.25 (q, C24), 23.69 (q, C25), 23.75 (q, C26), 21.81 (q, C27), 26.94 (q, C28), 28.45 (q, C29), 177.19 (s, C30), 51.37 (q, C31); HRMS: *m/z* calcd for C_31_H_46_O_4_: 484.6945; found: 484.3396.

**Methyl 2-hydroxymethylene-3,12-dioxo-18β*H*-olean-9(11)-en-30-oate (9):** Ethyl formate (3.75 mL, 39.5 mmol) and sodium methylate (2.1 g, 38.9 mmol) were added to a solution of ketone **8** (4.5 g, 9.3 mmol) in dry benzene (50 mL). The mixture was stirred at room temperature for 2 h. The reaction mixture was diluted with a mixture of chloroform/diethyl ether (1:3, 100 mL), and 5 % HCl was added to achieve pH<7. The organic layer was separated, and the aqueous layer was extracted with chloroform/diethyl ether (1:3, 3×50 mL). The combined organic layers were washed with saturated sodium hydrogen carbonate solution (3×50 mL), and brine (3×50 mL), and dried over magnesium sulfate. The solvent was removed to give an amorphous solid **9** (yield=4.5 g, 95 %). This material was used for the next reaction without further purification. An analytically pure sample was obtained by flash column chromatography (silica gel; hexane/ethyl acetate (9:1) followed by hexane/ethyl acetate (3:1)). ^1^H NMR (CDCl_3_): *δ*=0.90 (s, 3 H; C28-H_3_), 0.96 (s, 3 H; C27-H_3_), 1.09 (s, 3 H; C29-H_3_), 1.13 (s, 3 H; C24-H_3_), 1.15 (s, 3 H; C25-H_3_), 1.20 (s, 3 H; C23-H_3_), 1.38 (s, 3 H; C26-H_3_), 0.91 (m, H^16e^), 1.07 (m, H^15e^), 1.23 (dd, ^2^*J*(H^19a^,H^19e^)=13.3, ^3^*J*(H^19a^,H^18^)=13.3 Hz; H^19a^), 1.18–1.31 (m, 3 H; H^21a^, H^22e^, H^5a^), 1.49 (ddd, ^2^*J*(H^22a^,H^22e^)=14.0, ^3^*J*(H^22a^,H^21a^)=14.0, ^3^*J*(H^22a^,H^21e^)=4.1 Hz; H^22a^), 1.47–1.53 (m, H^7^), 1.59–1.67 (m, 3 H; 2 H^6^, H^7′^), 1.82 (m, H^15a^), 1.86 (m, H^16a^), 1.94 (dddd ^2^*J*(H^21e^,H^21a^)=13.3, ^3^*J*(H^21e^,H^22a^)=4.1, ^3^*J*(H^21e^,H^22e^)=3.4, ^4^*J*(H^21e^,H^19e^)=2.7 Hz; H^21e^), 1.99 (ddd, ^3^*J*(H^18^,H^19a^)=13.3, ^3^*J*(H^18^,H^13^)=4.6, ^3^*J*(H^18^,H^19e^)=3.3 Hz; H^18^), 2.21 (ddd, ^2^*J*(H^19e^,H^19a^)=13.3, ^3^*J*(H^19e^,H^18^)=3.3, ^4^*J*(H^19e^,H^21e^)=2.7 Hz; H^19e^), 2.26 (d, ^2^*J*(H^1^,H^1′^)=14.5 Hz; H^1^) and 2.58 (d, ^2^*J*(H^1′^,H^1^)=14.5 Hz; H^1′^)—AB-system, 3.02 (d, ^3^*J*(H^13^,H^18^)=4.6 Hz; H^13^), 3.71 (s, 3 H; OC31-H_3_), 5.90 (s, H^11^), 8.70 (d, ^3^*J*(H^32^_,_OH)=2.4 Hz; H^32^), 14.81 (d, ^3^*J* (OH,H^32^)=2.4 Hz; OH); ^13^C NMR (CDCl_3_): *δ*=36.85 (t, C1), 104.80 (s, C2), 188.08 (s, C3), 40.32 (s, C4), 48.07 (d, C5), 18.80 (t, C6), 31.25 (t, C7), 45.55 (s, C8), 175.36 (s, C9), 38.89 (s, C10), 124.37 (d, C11), 200.73 (s, C12), 47.86 (d, C13), 41.80 (s, C14), 26.22 (t, C15), 26.14 (t, C16), 31.93 (s, C17), 37.82 (d, C18), 33.74 (t, C19), 43.93 (s, C20), 31.14 (t, C21), 38.18 (t, C22), 28.14 (q, C23), 20.65 (q, C24), 23.36 (q, C25), 23.23 (q, C26), 21.82 (q, C27), 26.96 (q, C28), 28.48 (q, C29), 177.22 (s, C30), 51.42 (q, C31), 189.65 (d, C32); HRMS: *m/z* calcd for C_32_H_46_O_5_: 510.7046; found: 510.3345.

**Methyl 12-oxoisoxazolo[4,5-*b*]-18β*H*-olean-9(11)-en-30-oate (10):** Hydroxylamine hydrochloride (6.0 g, 86.0 mmol) was added to a solution of **9** (4.4 g, 8.6 mmol) in ethanol (120 mL) and water (12 mL). The mixture was heated under reflux for 2 h, cooled to room temperature, concentrated in vacuo, and water (100 mL) was added. The mixture was extracted with ethyl acetate (3×70 mL). The combined organic layers were washed with water (3×50 mL), brine (3×50 mL) and dried over magnesium sulfate. The solvent was evaporated and the solid was purified by column chromatography (silica gel; hexane/ethyl acetate (3:1)) to give **10** (yield=3.2 g, 73 %). ^1^H NMR (CDCl_3_): *δ*=0.91 (s, 3 H; C28-H_3_), 0.98 (s, 3 H; C27-H_3_), 1.10 (s, 3 H; C29-H_3_), 1.15 (s, 3 H; C25-H_3_), 1.24 (s, 3 H; C24-H_3_), 1.32 (s, 3 H; C23-H_3_), 1.40 (s, 3 H; C26-H_3_), 0.91 (m, H^16e^), 1.06–1.11 (m, H^15e^), 1.24 (dd, ^2^*J*(H^19a^,H^19e^)=13.2, ^3^*J*(H^19a^,H^18^)=13.2 Hz; H^19a^), 1.19–1.34 (m, 2 H; H^21a^, H^22e^), 1.44–1.57 (m, 3 H; H^*5a*^, H^*22a*^, H^*7*^), 1.64–1.79 (m, 3 H; 2 H^*6*^, H^*7′*^), 1.84 (m, H^15a^), 1.88 (m, H^16a^), 1.95 (dddd, ^2^*J*(H^21e^,H^21a^)=13.3, ^3^*J*(H^21e^,H^22a^)=4.2, ^3^*J*(H^21e^,H^22e^)=3.2, ^4^*J*(H^21e^,H^19e^)=2.7 Hz; H^21e^), 2.00 (ddd ^3^*J*(H^18^,H^19a^)=13.2, ^3^*J*(H^18^,H^13^)=4.6, ^3^*J*(H^18^,H^19e^)=3.2 Hz; H^18^), 2.21 (ddd, ^2^*J*(H^19e^,H^19a^)=13.2, ^3^*J*(H^19e^,H^18^)=3.2, ^4^*J*(H^19e^,H^21e^)=2.7 Hz; H^19e^), 2.38 (d, ^2^*J*(H^1^,H^1′^)=15.0 Hz; H^1^) and 2.75 (d, ^2^*J*(H^1′^,H^1^)=15.0 Hz; H^1′^)—*AB* system, 3.04 (d, ^3^*J*(H^13^,H^18^)=4.6 Hz; H^13^), 3.73 (s, 3 H; OC31-H_3_), 5.89 (s, H^11^), 8.04 (s, H^32^); ^13^C NMR (CDCl_3_): *δ*=33.42 (t, C1), 108.38 (s, C2), 171.94 (s, C3), 35.01 (s, C4), 49.53 (d, C5), 18.16 (t, C6), 31.24 (t, C7), 45.75 (s, C8), 175.87 (c, C9), 41.09 (s, C10), 124.65 (d, C11), 200.77 (s, C12), 47.90 (d, C13), 41.79 (s, C14), 26.27 (t, C15), 26.13 (t, C16), 31.95 (s, C17), 37.85 (d, C18), 33.79 (t, C19), 43.95 (s, C20), 31.16 (t, C21), 38.18 (t, C22), 28.65 (q, C23), 21.26 (q, C24), 24.52 (q, C25), 23.28 (q, C26), 21.87 (q, C27), 26.99 (q, C28), 28.51 (q, C29), 177.26 (s, C30), 51.47 (q, C31), 150.06 (d, C32); HRMS: *m/z* calcd for C_32_H_45_NO_4_: 507.7040; found: 507.3349.

**The mixture of tautomers (11):** Sodium methylate (11 g, 204 mmol) was added at 0°C to a solution of isoxazole **10** (3.0 g, 5.9 mmol) in methanol (85 mL) and diethyl ether (170 mL). The mixture was stirred at room temperature for 1 h. The resulting mixture was diluted with a mixture of chloroform/diethyl ether (1:3; 100 mL), and 5 % HCl was added to achieve pH<7. The organic layer was separated, and the aqueous layer was extracted with chloroform/diethyl ether (1:3; 3×50 mL). The combined organic layers were washed with saturated sodium hydrogen carbonate solution (3×50 mL) and brine (3×50 mL), and dried over magnesium sulfate. The solvent was removed to give a mixture of tautomers **11** (yield=3.0 g, 100 %). This material was used for the next reaction without further purification. HRMS: *m/z* calcd for C_32_H_45_NO_4_: 507.7040; found: 507.3349.

**Methyl 2-cyano-3,12-dioxo-18βH-olean-9(11),1(2)-dien-30-oate (12):** Mixture **11** (2.8 g, 5.5 mmol) and 2,3-dichloro-5,6-dicyano-1,4-benzoquinone (DDQ) (1.5 g, 6.5 mmol) in dry benzene (160 mL) were heated under reflux for 4 h. Insoluble matter was removed by filtration, and the filtrate was evaporated in a vacuum to give a solid. The solid was subjected to flash column chromatography (silica gel; benzene followed by benzene/acetone (10:1)) to give crude **12**. The crude product was purified by recrystallization from methanol/chloroform to give crystals **12** (yield 1.7 g, 61 %). M. p. 247–249°C; ^1^H NMR (CDCl_3_): *δ*=0.90 (s, 3 H; C28-H_3_), 0.96 (s, 3 H; C27-H_3_), 1.09 (s, 3 H; C29-H_3_), 1.14 (s, 3 H; C24-H_3_), 1.22 (s, 3 H; C23-H_3_), 1.44 C (s, 3 H; C26-H_3_), 1.47 (s, 3 H; C25-H_3_), 0.93 (dm, ^2^*J*(H^16e^,H^16a^)=13.3 Hz; H^16e^), 1.08 (m, H^15e^), 1.20 (dd, ^2^*J*(H^19a^,H^19e^)=13.2, ^3^*J*(H^19a^,H^18^)=13.2 Hz; H^19a^), 1.18–1.32 (m, 2 H; H^21a^, H^22e^), 1.48 (ddd, ^2^*J*(H^22a^,H^22e^)=14.0, ^3^*J*(H^22a^,H^21a^)=14.0, ^3^*J*(H^22a^,H^21e^)=4.2 Hz; H^22a^), 1.55 (dm, ^2^*J*(H^7e^,H^7a^)=13.5 Hz; H^7e^), 1.67–1.79 (m, 4 H; H^5a^, 2 H^6^, H^7a^), 1.82 (m, H^15a^), 1.87 (m, H^16a^), 1.94 (dddd, ^2^*J*(H^21e^,H^21a^)=13.3, ^3^*J*(H^21a^,H^22a^)=4.2, ^3^*J*(H^21e^,H^22e^)=3.2, ^4^*J*(H^21e^,H^19e^)=2.8 Hz; H^21e^), 2.02 (ddd, ^3^*J*(H^18^,H^19a^)=13.2, ^3^*J*(H^18^,H^13^)=4.7, ^3^*J*(H^18^,^19e^)=3.2 Hz; H^18^), 2.17 (ddd, ^2^*J*(H^19e^,H^19a^)=13.2, ^3^*J*(H^19e^,H^18^)=3.2, ^4^*J*(H^19e^,H^21e^)=2.8 Hz; H^19e^), 3.02 (d, ^3^*J*(H^13^,H^18^)=4.7 Hz; H^13^), 3.72 (s, 3 H; OC31-H_3_), 5.97 (s, H^11^), 8.01 (s, H^1^); ^13^C NMR (CDCl_3_): *δ*=165.65 (d, C1), 114.46 (s, C2), 196.42 (s, C3), 44.86 (s, C4), 47.57 (d, C5), 18.12 (t, C6), 31.58 (t, C7), 45.82 (s, C8), 168.18 (s, C9), 42.36 (s, C10), 124.17 (d, C11), 199.52 (s, C12), 48.04 (d, C13), 42.10 (s, C14), 26.03 (t, C15), 26.00 (t, C16), 31.88 (s, C17), 37.75 (d, C18), 33.61 (t, C19), 43.91 (s, C20), 31.11 (t, C21), 38.14 (t, C22), 26.88 (q, C23), 21.40 (q, C24), 26.61 (q, C25), 24.77 (q, C26), 21.81 (q, C27), 26.96 (q, C28), 28.46 (q, C29), 177.13 (s, C30), 51.48 (q, C31), 114.22 (s, C32); HRMS: *m/z* calcd for C_32_H_43_NO_4_: 505.6881; found: 505.3192.

**Cell culture and glycyrrhetinic acids derivatives:** Human KB-3-1 epidermoid carcinoma cell line, HeLa cervical epithelioid carcinoma cell line, MCF-7 breast adenocarcinoma cell line, SKNMC neuroblastoma cell line (Russian Cell Culture Collection, St. Petersburg), KB-8-5 multidrug resistant cancer cell line (kindly provided by Professor M. Gottesman (NIH, USA)), were cultured in DMEM supplemented with 10 % (*v*/*v*) heat-inactivated fetal bovine serum, penicillin (100 U mL^−1^; ICN Biomedicals, Inc), streptomycin (100 μg mL^−1^) and amphotericin (250 μg mL^−1^). Cells were maintained in a humidified atmosphere (5 % CO_2_, 37°C). The KB-8-5 cell line was incubated in the additional presence of vinblastine (300 nmoll^−1^).

Glycyrrhetinic acids derivatives were dissolved in DMSO (10 mmoll^−1^), and stock solution were stored at −20°C.

After treatments, both floating and adherent scraped cells were collected by centrifugation, and used for further analysis.

**Cell viability analysis by MTT assay**: Cancer cells, growing in log phase, were seeded in triplicate 96-well plates at a density of 5×10^3^ cells per well for HeLa cells, 7×10^3^ for KB-3-1, KB-8-5 and MCF-7 cells, and 30×10^3^ for SKNMC cells. The plates were incubated at 37°C in humidified 5 % CO_2_ atmosphere. Cells were allowed to adhere to the surface for 24 h, then treated with varying doses of the compounds for 24 h. Aliquots of [3-(4,5-dimethylthiazol-2-yl)-2,5-diphenyltetrazolium bromide] (MTT) solution (10 μL, 5 mg mL^−1^) were added to each well, and the incubation was continued for an additional 3 h. The dark blue formazan crystals (formed within healthy cells) were solubilized with DMSO, and the absorbance was measured at 570 nm in a Multiscan RC plate reader (Thermo LabSystems, Finland). The IC_50_ was determined as the compound concentration required to decrease the *A*_570_ to 50 % of the control (no compound, DMSO), and was determined by interpolation from dose-response curves.

**Analysis of antioxidant effect on the cytotoxicity of compound 12**: KB-3-1 cells growing in the log phase were seeded in triplicate in 96-well plates (7×10^3^ cells per well). The plates were incubated at 37°C in a humidified 5 % CO_2_ atmosphere. Cells were allowed to adhere to the surface for 24 h, then treated with GSH (1, 5, 15 or 45 mm) or with ascorbic acid (1, 3 or 5 mm), both alone and in combination with **12 (**1 μm). Cells were incubated with the compounds for 24 h and cell viability was analyzed by the MTT assay as described above.

**Morphological observation of nuclear change:** KB-3-1 cells were seeded into 24-well plates (10^5^ cells per well) containing glass cover slips. Cells were allowed to adhere to the surface for 24 h. Cells were treated with **12** (1 μm) or with DMSO (0.1 % (*v*/*v*)) for 6, 18 or 24 h at 37°C in a humidified 5 % CO_2_ atmosphere. After incubation, cells were fixed with 4 % formaldehyde for 15 min, and then stained for 30 min with Hoechst 33258 (200 ng mL^−1^). Cells were analyzed for the presence of fragmented nuclei and condensed chromatin by fluorescent microscopy.

**Apoptosis detection by Annexin V staining**: Log-phase KB-3-1 cells in six-well plates (5×10^5^ cells per well) were treated with **12** (0.3 μm or 1 μm) or with DMSO (0.1 % (*v*/*v*)) for 4, 18 or 24 h. The cells were stained with Annexin V-FITC and propidium iodide by using the ApopNexin-FITC apoptosis detection kit (Chemicon Millipore) according to the manufacturer's instructions. Briefly, cells were collected by scraping, washed twice with cold PBS, and centrifuged (400 *g*, 5 min). Cells were resuspended in binding buffer (1 mL) at a concentration of 1×10^6^ cells per mL, then a sample (200 μL) was transferred to a 5 mL culture tube, and Annexin V-FITC (3 μL) and 100×PI (2 μL) were added. Cells were incubated for 15 min at room temperature in the dark. Finally, binding buffer (300 μL) was added to each tube, and the quantity of apoptotic cells in samples was analyzed by flow cytometry (FC500, Beckman Coulter, USA). For each sample, 10 000 ungated events were acquired. Annexin V^+^/PI^−^ cells represented early apoptotic populations. Annexin V^+^/PI^+^ cells represented either late apoptotic or secondary necrotic populations.

**Mitochondria depolarization analysis:** Mitochondria involvement in apoptosis was measured by the mitochondrial depolarization that occurs early during the onset of apoptosis. KB-3-1 cells were treated with **1 (**1 μm), **12 (**1 μm) or DMSO (0.1 % (*v*/*v*)) for 6 h, and loss of mitochondrial potential was determined by using the mitochondrial potential sensor JC-1 (Molecular Probes, Invitrogen).

*Flow cytometry assay:* Cells were incubated for the appropriate time with the compounds, then collected, incubated in complete media in the dark with JC-1 (5 μg mL^−1^) at 37°C for 15 min, and washed with PBS. At the end of the incubation period the cells were washed twice with cold PBS, and resuspended in PBS (400 μL). J-aggregate and J-monomer fluorescence were recorded in the channesl 2 (FL2) and 1 (FL1), respectively, of an FC500 flow cytometer. Necrotic fragments were electronically gated out, on the basis of morphological characteristics on the forward light scatter versus side light scatter dot plot.

*Fluorescent microscopy assay:* Cells were plated into 24-well plates (10^5^ cells per well) containing glass cover slips, and allowed to adhere to the surface for 24 h. Cells were incubated for the appropriate time with the compounds. After incubation the cell culture media was removed and replaced with JC-1 reagent (5 μm) diluted in PBS. Cells were incubated at 37°C in a 5 % CO_2_ incubator for 15 min, and analyzed by fluorescence microscopy.

**Cytofluorimetric analysis of DNA content**: Exponentially growing KB-3-1 cells in 6-well plates (5×10^5^ cells per well) were treated with **12** (0.3 μm, or 1 μm) or DMSO (0.1 % (*v*/*v*)) for 18 h. After incubation, the cells were collected by centrifugation (400 *g*, 10 min), fixed with ice-cold 70 % ethanol for at least 1 h at 4°C and treated with RNase A from bovine pancreas (1 mg mL^−1^; Sigma) for 30 min at 37°C. PI (50 μg mL^−1^) was then added to the solution and the DNA content was quantitated by a flow cytometry. Cells in sub-G1 phase were considered apoptotic.

**Analysis of caspase activation:** After treatment of KB-3-1 cells with **1** (0.3 μm, 1 μm), or **12** (0.3 μm, 1 μm), or DMSO (0.1 % (*v*/*v*)) for 18 h, caspase activation was assayed by using the CaspACE FITC-VAD-FMK in situ marker (Promega).

*Flow cytometry assay:* Cells were incubated for the appropriate time in the presence of the compounds, collected, suspended in PBS (0.5 mL), and FITC-VAD-FMK (1 μL, 5 mm) was added. The cells were gently mixed and incubated for 20 min at RT in the dark. Cells were washed twice with PBS, and the pellets resuspended in PBS (0.5 mL). Flow cytometry was conducted within 10 min.

*Fluorescent microscopy assay:* Cells were seeded (10^5^ cells per well) into 24-well plates containing glass cover slips, and allowed to adhere to the surface for 24 h. After incubation for the appropriate time with the compounds, the cell culture medium was removed and replaced with JC-1 reagent (5 μm) diluted in PBS. Cells were incubated at 37°C in a 5 % CO_2_ incubator for 15 min. The cells were washed twice with PBS, and caspase activation was analyzed by fluorescence microscopy within 10 min.
